# Porcine NK Cells Stimulate Proliferation of Pseudorabies Virus-Experienced CD8^+^ and CD4^+^CD8^+^ T Cells

**DOI:** 10.3389/fimmu.2018.03188

**Published:** 2019-01-17

**Authors:** Steffi De Pelsmaeker, Bert Devriendt, Nick De Regge, Herman W. Favoreel

**Affiliations:** ^1^Laboratory of Immunology, Department of Virology, Parasitology and Immunology, Faculty of Veterinary Medicine, Ghent University, Merelbeke, Belgium; ^2^Department of Enzootic, Vector-Borne and Bee Diseases, Sciensano, Brussels, Belgium

**Keywords:** antigen presenting cells, natural killer cells, T cells, pig, pseudorabies virus

## Abstract

Natural killer (NK) cells belong to the innate immune system and play a central role in the defense against viral infections and cancer development, but also contribute to shaping adaptive immune responses. NK cells are particularly important in the first line defense against herpesviruses, including alphaherpesviruses. In addition to their ability to kill target cells and produce interferon-γ, porcine and human NK cell subsets have been reported to display features associated with professional antigen presenting cells (APC), although it is currently unclear whether NK cells may internalize debris of virus-infected cells and whether this APC-like activity of NK cells may stimulate proliferation of antiviral T cells. Here, using the porcine alphaherpesvirus pseudorabies virus (PRV), we show that vaccination of pigs with a live attenuated PRV vaccine strain triggers expression of MHC class II on porcine NK cells, that porcine NK cells can internalize debris from PRV-infected target cells, and that NK cells can stimulate proliferation of CD8^+^ and CD4^+^CD8^+^ PRV-experienced T cells. These results highlight the potential of targeting these NK cell features in future vaccination strategies.

## Introduction

Natural killer (NK) cells, members of the innate immune system, play an indispensable role in the defense against viral infections and cancer development, mainly because of their ability to kill virus-infected cells and malignant cells and their ability to produce cytokines, especially interferon-γ (IFNγ). NK cells are particularly important in the first line defense against herpesviruses, including alphaherpesviruses (1). Indeed, patients with NK cell deficiencies have been reported to suffer from aggravated and sometimes life-threatening alphaherpesvirus disease, including herpes simplex virus (HSV) encephalitis (2–5). The development of HSV vaccines has proven particularly cumbersome, and has not been successful yet (6, 7). Designing effective HSV vaccines may require new approaches, targeting previously underestimated elements of the immune response. It has been pointed out that the activity of NK cells may be an underappreciated facet of HSV vaccine design (8). Interestingly, for pseudorabies virus (PRV), a close relative of HSV in pigs, vaccination using live attenuated vaccines has been very successful (9–11).

We and others described that in addition to their well-known functions, porcine and human NK cell subsets also display features that are typically associated with professional antigen presenting cells [APCs; (12–14)]. Indeed, we recently found that, like human NK cell subsets, porcine NK cells show cell surface expression of MHC class II and the co-stimulatory molecules CD80/86 (14). In addition, using the NK-susceptible cell line K562, we found that porcine NK cells may take up debris of killed target cells and using an allogeneic T cell proliferation assay, we showed that porcine NK cells may trigger T cell proliferation (14). Interestingly, human NK cells have been suggested to directly present HSV antigen to T cells in recurrent herpetic lesions, thereby stimulating the antiviral T cell response (15). This has contributed to the idea that targeting NK cells in alphaherpesvirus vaccination may be particularly promising (15).

Thus far, it has not been investigated whether alphaherpesvirus vaccination may trigger expression of MHC class II and/or CD80/86 on NK cells, whether NK cells may take up debris of killed alphaherpesvirus-infected cells and whether indeed NK cells may stimulate proliferation of alphaherpesvirus-experienced T cells. Answers to these questions are of paramount importance to be able to address the potential of targeting these NK cell features in putative vaccination strategies. The current study aimed at clarifying these issues.

## Methods

### Vaccination Procedure

For primary vaccination, three 10-weeks-old pigs (crossbred female pigs derived from Rattlerow-Segher hybrid sows and Pietrain boars) received oronasal (4 × 10^7^ TCID_50_) and intramuscular (4 × 10^7^ TCID_50_) doses of live-attenuated PRV vaccine strain Begonia diluted in PBS. Three age-matched control pigs were vaccinated with PBS. Four weeks post primary vaccination, pigs received a booster vaccination, identical to the primary vaccination. Again, control pigs were vaccinated with PBS. The animal vaccination experiment and vaccination procedure were approved by the Ethical Committee of the Faculty of Veterinary Medicine (EC2017/119).

### Viruses

PRV NIA3 and Kaplan strains were used to infect swine kidney (SK) target cells. PRV Kaplan wild type virus was kindly provided by Thomas Mettenleiter (Friedrich Loeffler Institute). PRV NIA3 wild-type virus and NIA3-derived attenuated Begonia strain (gE-deleted and TK-negative, 10) were kindly provided by the ID-DLO, the Netherlands.

### Virus Neutralization Test

Blood was collected in Vacutest Kima gel and clot activator tube (Vacutest Kima, Arzergrande, Italy), following the manufacturer's instructions. Serum samples were heat-inactivated for 30 min at 56°C. For detection of PRV-specific antibodies a virus neutralization test was used, as described before (16). Briefly, a two times dilution series (1/2 to 1/256) was made in duplicate in Modified Eagle Medium in microtitre plates. Afterwards, 50 μl PRV dilution containing 100 plaque forming units PRV was added to the serum dilution and incubated for 1 h at 37°C. Thereafter, 100 μl Porcine Kidney 15 cell suspension in Modified Eagle Medium, supplemented with 10 % fetal calf serum, penicillin (1,000 U/ml), gentamicin (50 μg/ml) and fungizone (250 ng/ml), was added to each well followed by an incubation of 4 days at 37°C. Plates were then examined for the presence of plaques and the neutralizing titer of each serum sample was defined as the highest serial dilution that was capable of completely neutralizing the virus.

### Primary Porcine NK Cell Isolation and Culture

Heparinised blood samples (50 units/ml blood, LEO Pharma) were obtained from the external jugular vein of pigs (9–22 weeks old). The blood sampling procedure was approved by the Ethical Committee of the Faculty of Veterinary Medicine (EC2017/119 for vaccinated animals and EC2013/62 for healthy blood donors). Primary porcine NK cells were isolated from porcine peripheral blood mononuclear cells (PBMCs) by negative MACS depletion of CD3^+^ and CD172a^+^ cells followed by FACS purification using antibodies against porcine CD172a [IgG_1_, clone 74-22-15a, (17)], CD3 [IgG_1_, clone PPT3, (18)], and CD8α [IgG_2a_, clone 11/295/33, (19)] as described before (14). Antibodies were kindly provided by Dr. A. Saalmüller and produced in-house. Porcine NK cells were purity sorted, based on CD3^−^CD172a^−^CD8α^+^ expression using a BD FACS Aria III Cell Sorter (BD Biosciences), resulting in a ≥98% pure porcine NK population.

For the experiments where autologous T cells were needed, primary porcine NK cells were isolated from porcine PBMC by negative MACS depletion of CD6^+^ cells, by using anti-CD6 mAb [IgG_1_, clone a38b2; (20)] (see below) followed by FACS using the same antibodies as described above.

1 × 10^6^ primary porcine NK cells were cultured in 96-well flat bottomed plates (Nunc, Thermo Fisher Scientific) in RPMI (Gibco), supplemented with 10% (v/v) fetal calf serum (Thermo Fisher Scientific), 100 U/ml penicillin (Gibco), 100 μg/ml streptomycin (Gibco) (referred to as porcine NK medium). For cytolytic assays, porcine NK cells were primed with recombinant human (rh) IL-2 (20 ng/ml) for 16–18 h before the assay. For T cell proliferation experiments, porcine NK cells were primed with WT PRV NIA3 strain (5 × 10^7^ TCID_50_/ml) for 16–18 h before the assay.

### Primary CD6^+^ T Cell Isolation

Porcine CD6^+^ T cell isolation was performed as described before (14, 21). CD6^+^ T cells were enriched from PBMCs to a purity of >95% by positive immunomagnetic selection with the anti-CD6 mAb [IgG_1_, clone a38b2; (20)] and goat anti-mouse IgG_1_ microbeads and using LS columns.

### Cell Surface Expression Analysis via Flow Cytometry

For flow cytometric analysis, cells were washed in PBS. All incubation steps were performed in 96-well conical bottomed plates for 40 min at 4°C. The different combinations of primary monoclonal antibodies (mAbs) and secondary reagents used for each assay are listed in Table 1 and were diluted in PBS. Sytox blue (Thermo Fisher Scientific) was used to discriminate live and dead cells for the APC phenotyping of NK cells. Flow cytometry was performed using a BD FACS Aria III (BD Biosciences), and samples were analyzed with FACSDiva software (BD Biosciences) and FlowJo software (doublet discrimination). Minimum 10,000 NK cells were analyzed for the APC phenotyping, minimum 20,000 T cells in the recall PBMC assay and minimum 1,000 T cells/subset were analyzed in the NK/T proliferation experiments.

**Table 1:** Primary and secondary antibodies used for cell surface expression analysis by flow cytometry.

**Antigen**	**Clone**	**Isotype/recombinant**	**Labeling strategy**
**APC PHENOTYPING OF NK CELLS**			
CD3 (18)	PPT3	IgG_1_	Secondary antibody R-phycoerythrin conjugated goat anti-mouse IgG_1_
CD172a (17)	74-22-15a	IgG_1_	Secondary antibody R-phycoerythrin conjugated goat anti-mouse IgG_1_
CD8α (19)	11/295/33	IgG_2a_	Biotin-streptavidin Allophycocyanin
CD80/86		Recombinant human CD152 [CTLA-4]-muIg fusion protein mouse IgG_2a_ (Ancell, Bayport, MN)	Directly labeled Fluorescein isothiocyanate conjugated
MHC II (Bio-Rad)	2E9/13	IgG_2b_	Secondary antibody Alexa fluor 488 conjugated goat anti-mouse IgG_2b_
**PROLIFERATION ASSAYS**			
CD3 (18)	PPT3	IgG_1_	Secondary antibody R-phycoerythrin conjugated goat anti-mouse IgG_1_
CD8α (19)	11/295/33	IgG_2a_	Secondary antibody Alexa Fluor 647 conjugated goat-anti-mouse IgG_2a_
CD4 (17)	74-12-4	IgG_2b_	Secondary antibody Fluorescein isothiocyanate conjugated goat anti-mouse IgG_2b_

### Infections of Swine Kidney (SK) Cells

SK cells were used as target cells in cytolytic assays and were cultivated in Modified Eagle Medium (Life Technologies, Thermo Fisher Scientific) supplemented with 10% (v/v) fetal calf serum, 100 U/ml penicillin, 100 μg/ml streptomycin and 0.05 mg/ml gentamycin. SK cells were detached from cell culture flasks (175 cm^2^) using trypsin, seeded in suspension culture flasks (25 cm^2^, Sarstedt, Nümbrecht, Germany) at 1.2 × 10^7^ cells/8.5 mL, inoculated at a multiplicity of infection of 10, and put on a rocking platform at 37°C for 12 h.

### Cytolytic and Internalization Assays

Mock or PRV wild type NIA3- or wild type Kaplan-infected SK cells were labeled with carboxyfluorescein succinimidyl ester (CFSE) proliferation dye (CellTrace™ CFSE cell proliferation kit; Invitrogen, Thermo Fisher Scientific) according to the manufacturer's recommendations. Briefly, 1.0 × 10^6^ SK cells/ml were resuspended in porcine NK medium with 5 μM CFSE and incubated during 15 min at 37°C. The labeling reaction was stopped by addition of ice-cold porcine NK medium. Afterwards, CFSE-labeled SK cells were washed in porcine NK medium to remove excess CFSE. SK cells were co-incubated with IL-2-primed NK cells at an effector:target ratio of 25:1 for 2–8 h (as indicated in the text) at 37°C. For internalization assays, subsequently, cells were washed 2 times with 1 ml PBS, fixed with 3% paraformaldehyde (PFA), washed 2 times with 1 ml PBS, incubated for 40 min at 37°C with anti-CD16 mAb (IgG_1_, clone G7, AbD Serotec), washed 2 times with 1 ml PBS, incubated for 40 min at 37°C with goat anti-mouse IgG_1_ Alexa Fluor 647 (Thermo Fisher Scientific) and washed 2 times with 1 ml PBS. Flow cytometry was performed using a BD FACS Aria III, and samples were analyzed with FACSDiva software and FlowJo software (doublet discrimination).

For cytolytic assays, after co-incubation at an effector:target ratio of 20:1 for 4 h, viability of 5,000 target cells was assessed by propidium iodide and flow cytometry. The percentage of NK-mediated lysis was calculated using the formula (% dead target_NK_ – % dead target_spont_)/(%dead target_maximum_ – % dead target_spont_).

### Proliferation Assays

Isolated PBMCs or primary CD6^+^ T cells were labeled with violet proliferation dye (CellTrace™ Violet cell proliferation kit; Life Technologies, Thermo Fisher Scientific) according to the manufacturer's recommendations. Briefly, 1.0 × 10^6^ CD6^+^ T cells or PBMCs/ml were resuspended in PBS with 5 μM violet dye and incubated for 20 min at 37°C. The labeling reaction was stopped by addition of ice-cold mixed leukocyte reaction medium, consisting of DMEM (Life Technologies, Thermo Fisher Scientific), supplemented with 10% (v/v) fetal calf serum, 100 U/ml penicillin and 100 μg/ml streptomycin. Excess dye was removed by two mixed leukocyte reaction medium washing steps. Thereafter, for the recall PBMC assay, 1 × 10^6^ PBMCs were cultured in a 96-well flat-bottomed plate with PRV WT NIA3 (1 × 10^7^ TCID_50_) for 4 days.

For the T/NK proliferation experiments, 4 weeks post-booster vaccination, PRV WT NIA3-stimulated NK cells (1.2 × 10^5^) were co-cultured in a round bottomed plate with medium, mock- or PRV WT NIA3-infected target SK cells at an effector:target ratio of 20:1, followed by adding 1.2 × 10^5^ violet dye-labeled CD6^+^ T cells per well after 2 h co-incubation, during 4 days in the presence of rh IL-2 (20 ng/ml)(added on the first day).

### Statistical Analysis

Statistical analysis was performed using Graphpad Prism 5. Data were analyzed for statistical differences with an unpaired *t*-test at the 5% significance level to compare two conditions. To compare more than two conditions, data were analyzed for statistical differences with a repeated measures analysis of variance (ANOVA) at the 5% significance level. *Post-hoc* comparisons between different conditions were performed using Tukey's range test.

## Results

### Porcine NK Cells Internalize Debris Derived From Killed PRV-Infected Target Cells

Recently, using the NK-susceptible cell line K562, we showed that porcine NK cells are able to perform actin polymerization-dependent internalization of cell debris derived from their killed target cells (14). Here, we investigated whether porcine NK cells may also internalize debris from killed PRV-infected target cells, which is an important prerequisite for potential antigen presenting properties of porcine NK cells in the context of an alphaherpesvirus infection.

To test this, primary porcine NK cells of healthy blood donors were used in cytolytic assays using CFSE-labeled mock-infected and wild type (WT) PRV-infected swine kidney (SK) target cells. SK cells were infected at a MOI of 10 which we showed earlier to result in a 100% infection rate (22). Infection rate was confirmed for each assay by cell surface staining of viral protein gD and flow cytometric analysis and was always 100% (data not shown). Earlier, we also have shown that co-incubation of NK cells with PRV-infected or mock-infected SK cells leads to preferential killing of PRV-infected SK cells compared to mock-infected cells (23, 24).

At different time points post co-incubation of NK and target cells, NK cells were analyzed by flow cytometry for CFSE fluorescence as an indication for internalization of target cell debris, as described earlier for killed K562 target cells (14). To ensure that NK cells do not take up free CFSE from lysed target cells which has not covalently bound to cellular proteins, a control experiment was performed where NK cells were incubated for 2 h with either CFSE-labeled K562 cells or with supernatant of CFSE-labeled K562 cells that had been incubated before for 2 h with NK cells to trigger K562 cell killing. NK cells incubated with supernatant of killed CFSE-labeled K562 cells did not become CFSE positive (Supplemental Figure 1).

**Figure 1:**
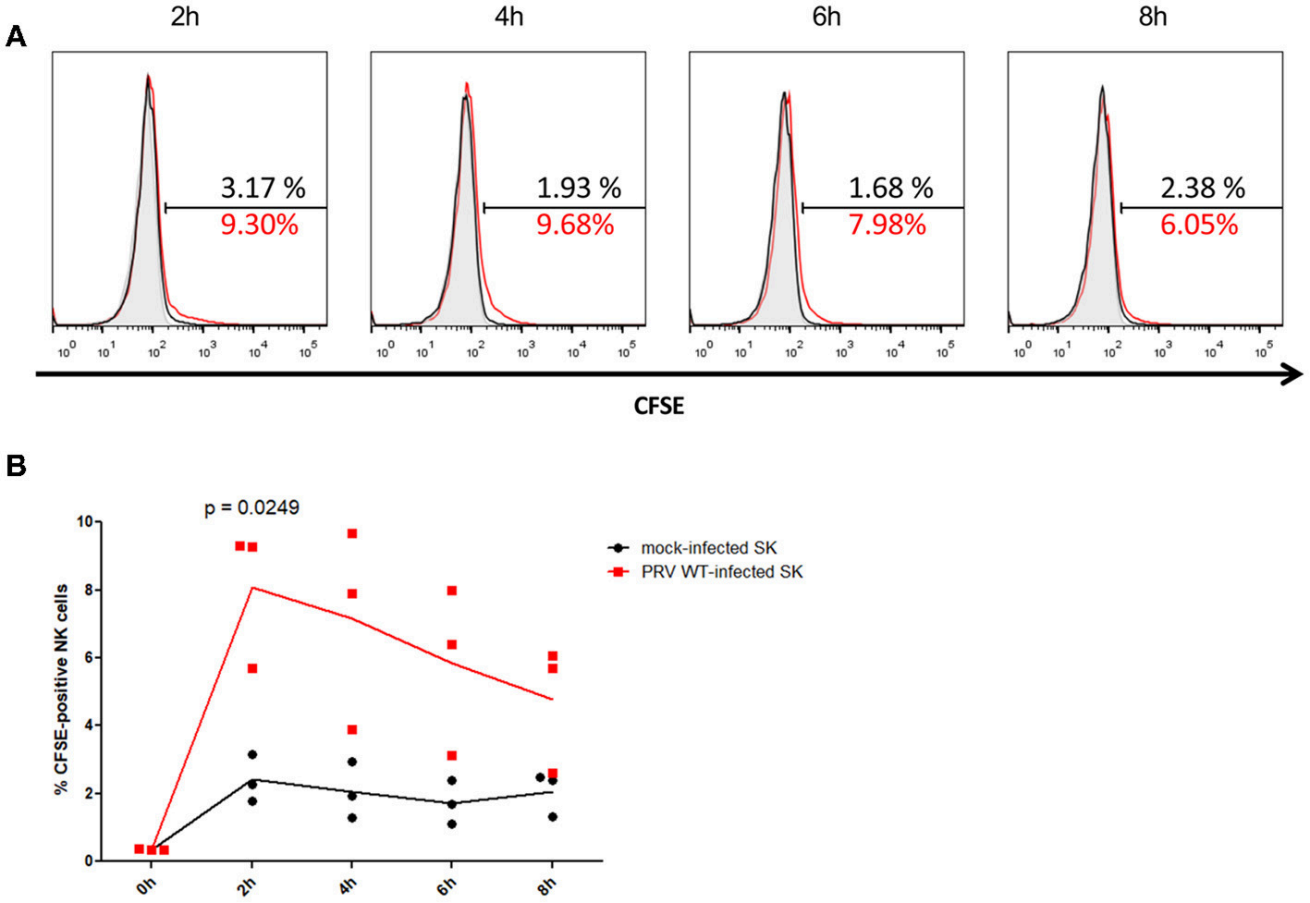
Porcine NK cells internalize fragments of killed PRV-infected target cells. **(A)** Histograms show the CFSE signal of IL-2-primed NK cells that were incubated for the indicated times with PRV WT-infected SK cells (NK:target ratio 25:1) that had been labeled with CFSE (red open histogram), CFSE-labeled mock-infected SK cells (black open histogram) or not incubated with target cells (gray shaded histogram) of one representative pig (out of three). The amount of CFSE-positive cells (%) is indicated in the histograms. **(B)** Graph shows the amount of CFSE-positive IL-2-primed NK cells that were co-incubated for the indicated times with CFSE-labeled mock-infected SK cells or PRV wild type-infected SK cells (effector target ratio of 25:1). Dot plot shows the results of three individual blood donors and the mean values are connected with a line. *P*-value threshold for statistically significant differences was 0.05, *p*-values are indicated in the graphs.

After 2 h of co-incubation of NK cells with CFSE-labeled PRV-infected or mock-infected SK cells, a statistically significant higher amount (mean ± SD) (8.1 ± 2.1%) of CFSE-positive NK cells were detected upon co-incubation with PRV-infected target cells compared to co-incubation with mock-infected cells (2.4 ± 0.7%), indicative for internalization of debris derived from PRV-infected target cells by the NK cells (Figure 1). This increase in the number of CFSE-positive NK cells was followed by a gradual decrease (from 7.2 ± 3.0% at 4 h to 4.7 ± 1.9% at 8 h) (Figure 1), all in line with earlier results in K562 cells (14), suggesting that NK cells are able internalize debris and further process the internalized debris of PRV-infected target cells.

In conclusion, porcine NK cells can internalize antigens derived from PRV-infected target cells, suggesting they may be able to present PRV antigens to T cells.

### APC Markers on the Surface of Porcine NK Cells Are Upregulated After Primary PRV Vaccination but Not After Booster Vaccination

**Figure 2:**
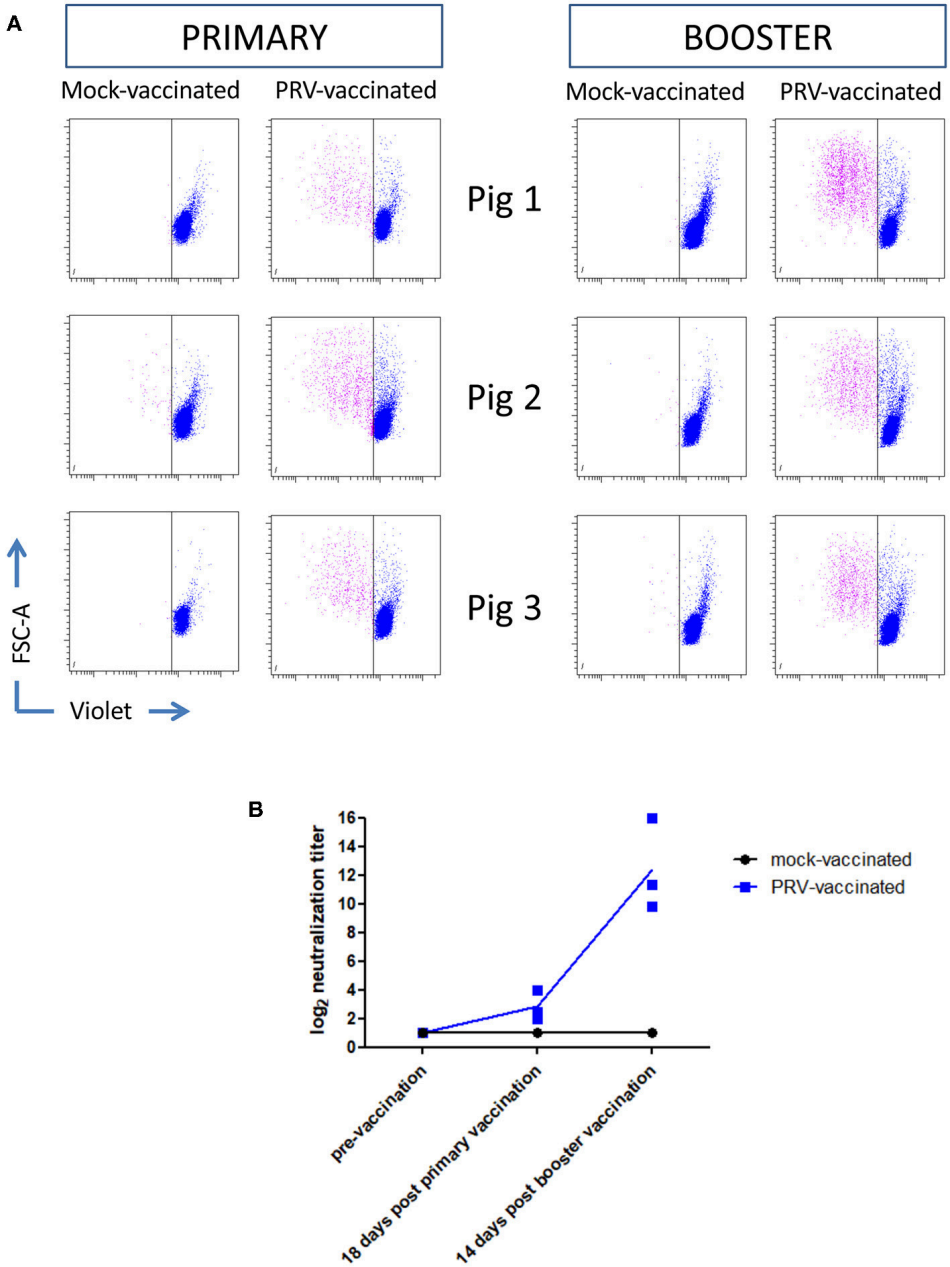
PRV vaccination induces PRV-experienced T cells and seroconversion. **(A)** Flow cytometry analysis of violet labeled PRV-stimulated PBMC isolated from mock-vaccinated pigs (*n* = 3) and PRV-vaccinated animals (*n* = 3) at 18 and 14 days post primary and booster vaccination, respectively, is shown. Dot plots show the proliferation-induced dilution of the violet level of the CD3^+^ T cell fraction after 4 days. **(B)** Seroconversion in mock-vaccinated and PRV-vaccinated pigs was tested by a virus neuralization assay at different timepoints: pre-vaccination, 18 days post primary vaccination, and 14 days post booster vaccination. The log equivalent of the serum dilution able to neutralize 100 PFU PRV is reported as the neutralization titer. Dot plot shows the results of the three animals in each group (mock- and PRV-vaccinated) and the mean values are connected with a line.

Ten-week-old pigs were vaccinated with the live attenuated PRV vaccine strain Begonia followed by a booster vaccination 4 weeks later. Mock-vaccinated animals (vaccinated with PBS) served as controls. Figures 2A,B show that all PRV-vaccinated, but not the mock-vaccinated animals, developed PRV-specific serum antibodies and T cell responses based on recall PBMC assays, indicating successful vaccination. Serum antibodies and T cell responses could already be detected after primary vaccination, and were increased upon booster vaccination.

**Figure 3:**
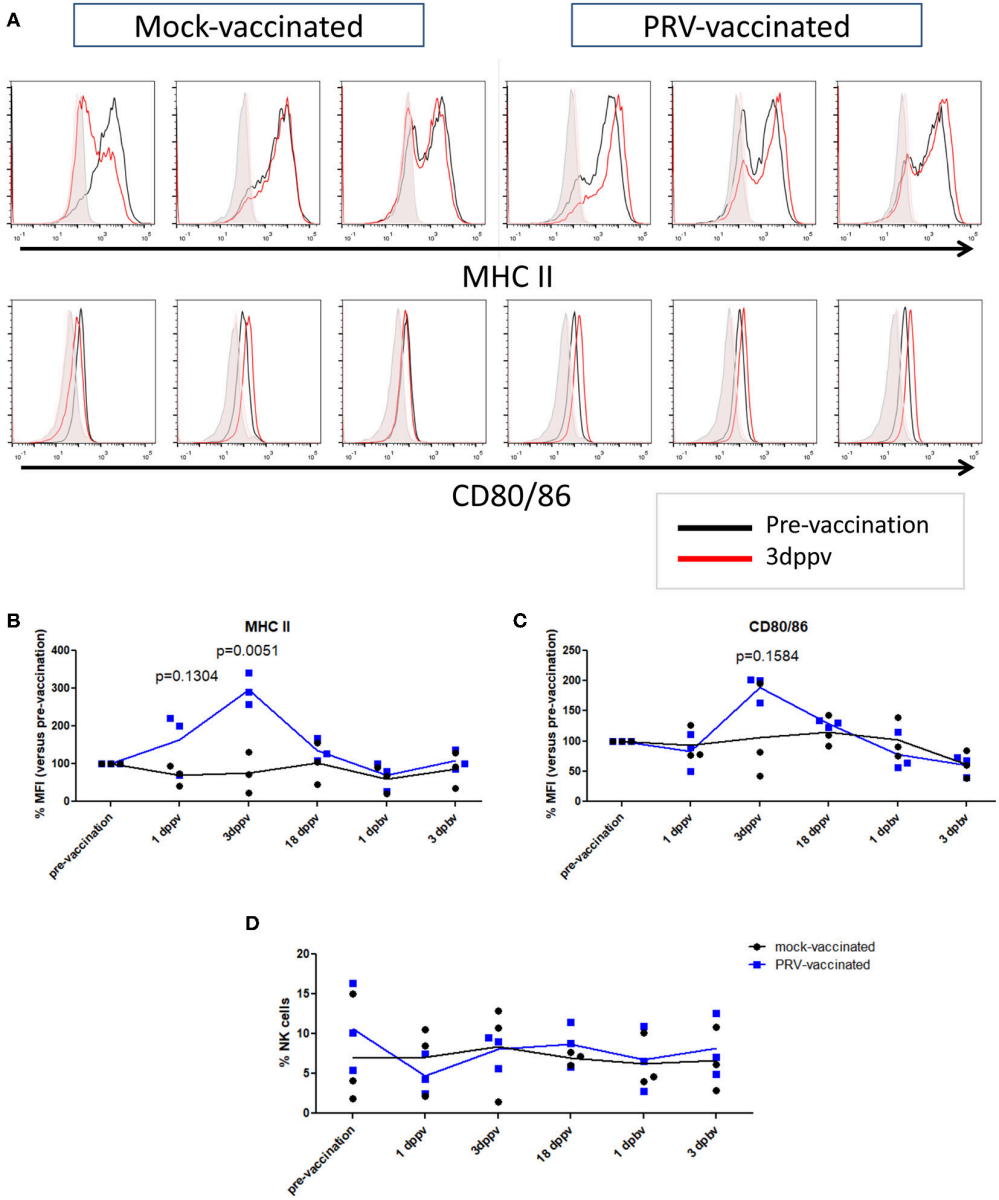
MHC II and CD80/86 expression on porcine NK cells before vaccination and after primary and booster vaccination. **(A)** Histograms show MHC II (upper panel) and CD80/86 (lower panel) expression on NK cells within the PBMC population before vaccination (pre-vaccination) and 3 days post primary vaccination (dppv). An overlay of the signals (pre-vaccination: black open histogram; 3 dppv: red open histogram) and isotype controls (pre-vaccination: gray shaded histogram; 3 dppv: red shaded histogram) of three mock-vaccinated animals (left) and three PRV-vaccinated animals (right) is shown. Graphs show MFI values of MHC II **(B)** and CD80/86 **(C)** expression on porcine NK cells before vaccination, 1, 3, and 18 days post primary vaccination and 1 and 3 days post booster vaccination. The MFI value of the time point before vaccination was set to 100% and the following time points were compared with the time point before vaccination. **(D)** Dot plot shows the percentage of NK cells within the PBMC population before vaccination, 1, 3, and 18 days post primary vaccination and 1 and 3 days post booster vaccination. Dot plot shows the results of three animals of each group (mock-vaccinated and PRV-vaccinated group) and the mean values are connected with a line. *P*-value threshold for statistically significant differences was 0.05, *p*-values are indicated in the graphs (dppv, day(s) post-primary vaccination; dpbv, day(s) post-booster vaccination).

In a next step, we investigated whether the APC phenotype of NK cells is affected upon vaccination of pigs with a live attenuated vaccine. At different time points (3 days before vaccination, 1, 3, and 18 days post primary vaccination, and 1 and 3 days post booster vaccination), PBMCs were isolated and NK cells within the PBMC population from mock- and PRV-vaccinated animals were tested for their surface expression of the APC markers MHC class II and CD80/86 (Figures 3A–C). NK cells were gated based on their CD3^−^CD172a^−^CD8α^+^ expression profile. Data are shown as the median fluorescence intensity (MFI) value of each time point for each animal compared to the MFI before vaccination (pre-vaccination—set to 100%) for this animal. Figure 3B shows a statistically significant upregulation of MHC class II expression on the NK cell surface at 3 days after primary vaccination (Figure 3B), which was not observed in the control animals. The MHC class II upregulation was associated with a cell surface upregulation of CD80/86 expression in the PRV-vaccinated group at 3 days post primary vaccination, although this upregulation was not statistically significant (Figure 3C). This upregulation of cell surface MHC class II (and CD80/86) on NK cells was only observed early after primary vaccination, and not later post primary vaccination (18 days) or after booster vaccination (Figures 3B,C).

We also tested whether the percentage of NK cells in blood changes after PRV vaccination. Although substantial individual variability in NK cell percentage in blood could be observed over time, no obvious differences or trends could be observed over time in either PRV- or mock-vaccinated animals (Figure 3D).

All together, these data suggest that the APC cell surface markers of NK cells are triggered early upon primary vaccination against the porcine alphaherpesvirus PRV, but not after booster vaccination with the same vaccine.

### PRV Primed Porcine NK Cells Stimulate T Cell Proliferation of PRV-Experienced CD8^+^ and CD4^+^CD8^+^ T Cells Upon Killing of PRV-Infected Target Cells

**Figure 4:**
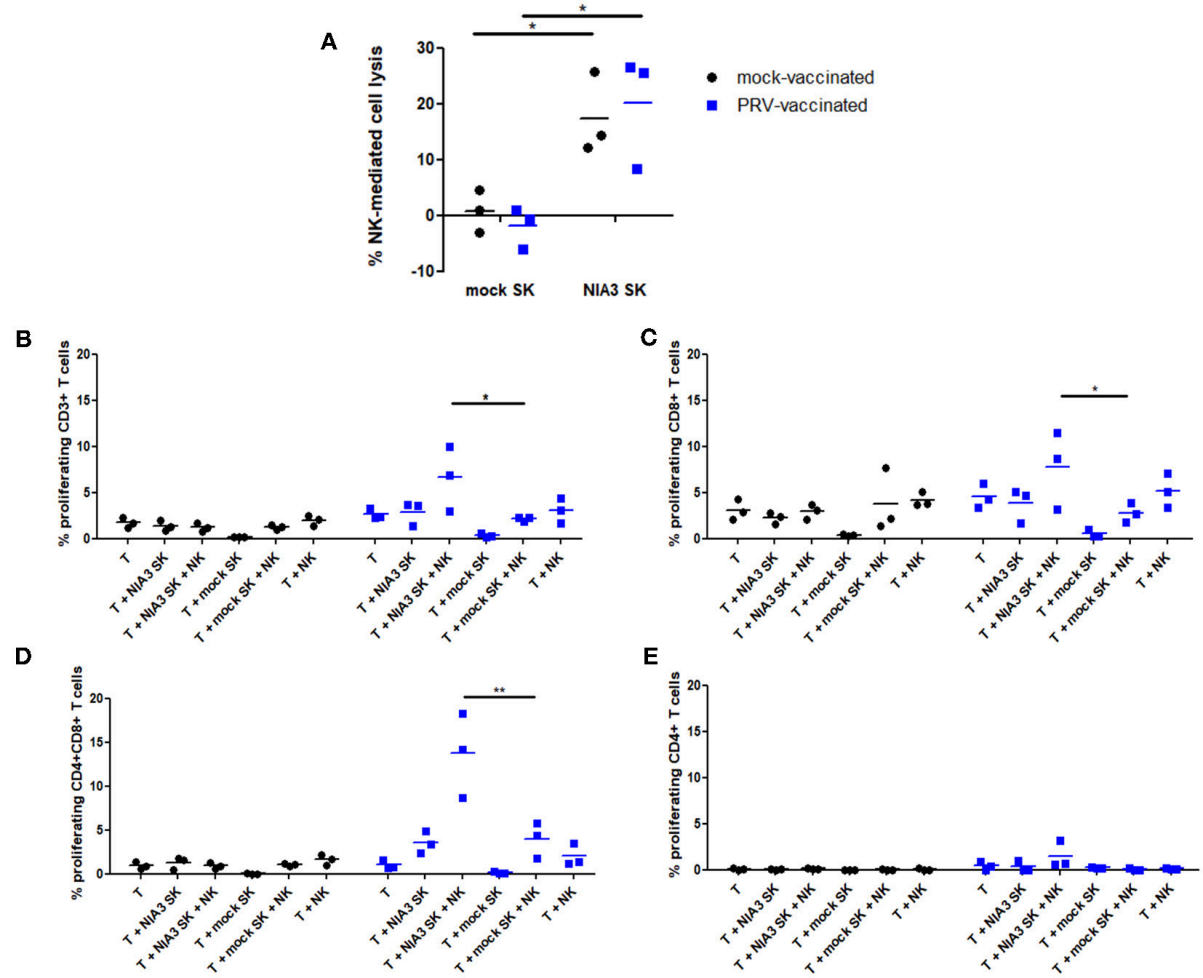
NK cells from PRV-vaccinated pigs stimulate T cell proliferation of PRV-experienced CD8^+^ and CD4^+^CD8^+^ T cells after killing of PRV-infected target cells. **(A)** SK cells were mock-infected or PRV-infected and subsequently incubated with NK cells from mock- or PRV-vaccinated animals for 4 h at a effector:target ratio of 20:1. Viability of target cells was assessed by propidium iodide and flow cytometry. Dot plots show the results of the three animals in each group (mock- and PRV-vaccinated) and the mean values are indicated with a horizontal line. **(B–D)** PRV-stimulated NK cells were either or not co-cultured with medium, mock- or PRV-infected target SK cells at an effector:target ratio of 20:1. After 2 h co-incubation, violet dye-labeled CD6^+^ T cells were added and incubated for 4 days. Dot plots show the T cell proliferation of CD3^+^ **(B)**, CD8^+^ **(C)** CD4^+^CD8^+^ **(D)**, and CD4^+^ **(E)** T cells of the three animals in each group (left panels: mock-vaccinated animals, right panels: PRV-vaccinated animals). The mean values are indicated with a horizontal line. Statistically significant differences in T cell proliferation between NK cells that were pre-incubated with PRV-infected SK target cells vs. mock-infected SK target cells are indicated with asterisks (^*^p < 0.05; ^**^p < 0.01).

The data above suggest that in particular circumstances, porcine NK cells may have the potential to stimulate PRV-specific T cells during PRV vaccination/infection. Hence, we investigated whether NK cells, upon killing of PRV-infected target cells, may be able to stimulate proliferation of PRV-experienced T cells. Since 2 h co-incubation of NK cells with PRV-infected cells leads to maximal internalization of target cell debris [Figure 1 and (14)], NK cells derived from blood of PRV-vaccinated or mock-vaccinated animals were incubated with PRV-infected or mock-infected SK cells for 2 h before addition of T cells. In line with earlier findings (23, 24), PRV-infected SK cells show increased susceptibility to NK cell-mediated lysis, compared to mock-infected SK cells (Figure 4A). As a control, we confirmed that the killing efficiency of PRV-infected SK cells was similar for PRV-stimulated NK cells derived from either PRV-vaccinated or mock-vaccinated animals (Figure 4A). At 2 h post incubation of NK cells with PRV- or mock-infected SK cells, autologous T cells were added to the co-culture for 4 days and T cell proliferation was determined. Conditions with T cells alone, T cells co-incubated with mock/PRV-infected SK cells but without NK cells and T cells co-incubated with NK cells but without SK cells served as controls.

Proliferation of CD3^+^ T cells was very low to virtually absent in all control conditions: T cells alone, T cells in the presence of mock- or PRV-infected SK cells alone or T cells in the presence of NK cells and mock-infected SK target cells. A statistically significant NK cell-dependent increase in CD3^+^ T cell proliferation could be observed in the condition where NK cells were able to kill PRV-infected SK cells, but only when T cells were derived from blood of PRV-vaccinated pigs, but not when T cells were derived from PRV-naïve (mock-vaccinated) animals (Figure 4B). This indicates that NK cells that are allowed to internalize debris from PRV-infected SK target cells are able to stimulate proliferation of PRV-experienced T cells.

*In vitro* proliferation of PRV-experienced T cells in PRV-primed mixed leukocyte assays has been shown to result in the proliferation of CD8^+^ and CD4^+^CD8^+^ double positive (DP) T cell fractions (25). The latter population is of particular importance in pigs, as it can trigger memory responses (26, 27). Therefore, we analyzed whether porcine NK cells can contribute to the proliferation of these two specific populations. Supplemental Figure 2 shows the gating strategy to discriminate the different T cell subsets.

Both CD8^+^ and CD4^+^CD8^+^ DP T cell fractions showed a significantly increased NK cell-dependent proliferation when using PRV-infected SK target cells and T cells from PRV-vaccinated animals, when compared to using mock-infected SK target cells (Figures 4C,D). No increase in CD4^+^ T cell proliferation was observed in these conditions (Figure 4E). Violet proliferation dot plots of T cells of one PRV-vaccinated animal are shown in Supplemental Figure 3.

In conclusion, these data show that upon killing of PRV-infected target cells, porcine NK cells are able to stimulate proliferation of PRV-experienced T cells, particularly the CD8^+^ and CD4^+^CD8^+^ DP T cell subpopulation.

## Discussion

NK cells are best known for their ability to kill malignant or viral infected cells, but they have also the capacity to stimulate the adaptive immune response. On the one hand, they can produce IFNγ upon activation, thereby steering the adaptive immune response toward a Th1 response. On the other hand, there is evidence that, at least in human and pig, NK cell subsets can perform activities that resemble those of professional APCs. Our data show that MHC class II (and CD80/86) expression on the surface of porcine NK cells is increased shortly after primary vaccination with an attenuated PRV vaccine strain, and that NK cells can use this APC-like activity to stimulate proliferation of PRV-experienced T cells.

We found that shortly after a primary PRV vaccination of pigs, at 3 days post vaccination, NK cells in blood showed an increased cell surface expression of MHC class II. These data are in line with data on HSV, where it has been shown that *in vitro* addition of viral antigens to NK cells triggers increased expression of MHC class II (15). In our assays, MHC class II upregulation was associated with an increase, although not statistically significant, in cell surface expression of the co-stimulatory CD80/86 molecules.

The significant increase in MHC class II expression (and non-significant trend of increased CD80/86 expression) observed early after primary vaccination faded at later time points (18 days post vaccination). A speculative explanation for this observation is that after vaccination, the stimulated NK cells may migrate to the infected tissues and/or to secondary lymphoid organs where the antigen-bearing NK cells could settle in the T-cell area where they may stimulate T cells. It will be interesting in further studies to analyze early after vaccination whether MHC class II-expressing NK cells can be observed in the vaccinated tissue and/or in the T-cell areas in the draining lymph nodes. The increase in MHC class II (and trend of increased CD80/86 expression) was observed early after primary vaccination but not after booster vaccination. One speculative explanation for this observation may be that at the time of booster vaccination, neutralizing antibodies and/or T cells can limit virus replication, resulting in a lower number of viral particles to stimulate increased expression of MHC II (and/or CD80/86) on NK cells. Indeed, we found that primary vaccination was sufficient to induce a PRV-specific T cell response and PRV-specific serum antibodies (Figure 2). Alternatively at the time of booster vaccination, it is possible, and even likely, that PRV-experienced memory T cells are present in the different tissues that were subjected to vaccination (respiratory tract and skin). Hence, we cannot exclude that booster vaccination may also have stimulated APC-like features in local NK cells, but that these cells remained present in the tissues where they may have stimulated the pre-existing tissue-resident PRV-specific memory T cells. In support of the idea that NK cells may stimulate pre-existing tissue-resident memory T cell responses against alphaherpesviruses, for HSV, it has been found that a large proportion of NK cells (57%) in human recurrent herpetic lesions were in contact with CD4^+^ T cells and were suggested to present antigen to these T cells (15).

We showed that upon killing of PRV-infected target cells, porcine NK cells are able to stimulate proliferation of CD8^+^ and CD4^+^CD8^+^ PRV-experienced T cells, especially the CD4^+^CD8^+^ DP T cell subpopulation (Figure 4). Proliferation of CD8^+^ T lymphocytes may directly contribute to the elimination of PRV-infected cells, since Zuckermann et al. (27) already have shown that porcine CD8^+^ T lymphocytes can kill PRV-infected target cells (27). Proliferation of the CD4^+^CD8^+^ DP T cell subset is particularly interesting since this special T cell population in pigs possesses properties of mature antigen-experienced cells, induced by stimulation with recall antigen (28), and can have a memory function (26, 27). This T cell subpopulation has also been shown to contribute to the production of virus-specific antibodies against PRV by B cells (29) and appears to display a MHC class II-restricted cytolytic function upon stimulation with PRV (30). Not only for PRV, but also for porcine rubulavirus infection, CD4^+^CD8^+^ cells have been reported and may participate in the defense against virus infection (31). Since CD4^+^CD8^+^ T cells have been reported to arise from CD4^+^ T cells that can acquire CD8 expression after antigen encounter (28, 31), future research aimed at vaccination purposes will have to show whether porcine NK cells may not only stimulate expansion of this PRV-experienced CD4^+^CD8^+^ memory T cell population upon re-encounter and processing of the same antigen, but also if NK cells can contribute to the priming of naïve CD4^+^ T-cells.

Of note, both cytolytic CD8^+^ and antigen-experienced CD4^+^CD8^+^ porcine T cell subsets have been reported to express MHC II (32–35) A theoretical possibility could be that the T cells themselves may take up antigen released from infected cells and present it to each other, thereby contributing to the T cell proliferation observed in our assays. However, porcine T cells have not been reported to internalize or present antigen. Since we showed earlier that PRV causes substantial lysis of infected SK cells at 24 hpi, reaching 100% lysis at 48 hpi (22), the 4 day proliferation assays performed in the current study would allow sufficient time for T cells to internalize and present antigen. However, we found no evidence for T cell proliferation when T cells were added to PRV-infected SK cells in the absence of NK cells in the 4 day proliferation assays, whereas significant T cell proliferation could be observed in the presence of NK cells (Figures 4B–D), arguing against the hypothesis of T cell presentation of PRV antigens in these assays.

Human NK cells have been suggested to present antigen to and stimulate T cell responses against HSV and several other reports indicate that some human NK cell subsets may express MHC II and co-stimulatory molecules that stimulate T cell proliferation (12, 13, 15, 36–40). In this context, our data are the first to directly show that NK cells indeed can stimulate proliferation of alphaherpesvirus-experienced T cells upon killing of alphaherpesvirus-infected target cells. These data underline the notion that the pig may be a particularly interesting model to address this less-well known aspect of NK cell biology. In contrast to human and porcine NK cells, murine NK cells do not express MHC class II or co-stimulatory molecules, but can acquire these molecules from dendritic cells (DCs) via a membrane exchange process called trogocytosis (41). Possibly because of the low quantities of MHC class II and co-stimulatory molecules that decorate the NK cell surface upon trogocytosis, MHC class II and co-stimulatory molecules on mouse NK cells have been associated with suppression of DC-induced CD4^+^ T-cell responses (41), which is very different from the stimulation of T cell proliferation observed in man and pig.

The results obtained in this study highlight the potential of using the porcine model as a complementary tool to further investigate the role of innate immune players, like NK cells, and underestimated features of these cells in the context of alphaherpesvirus infection to provide key insights in the development of successful (alphaherpesvirus) vaccines.

## Author Contributions

SD performed the experiments. BD and ND provided reagents. SD and HF designed the research, made the figures, and wrote the manuscript. SD, HF, ND, and BD, analyzed the results.

### Conflict of Interest Statement

The authors declare that the research was conducted in the absence of any commercial or financial relationships that could be construed as a potential conflict of interest.

## Abbreviations

DP: double positive
PBMC: peripheral blood mononuclear cell
PRV: pseudorabies virus
SK: Swine Kidney
WT: wild type.
