# Supplementary material for: Porcine NK Cells Stimulate Proliferation of Pseudorabies Virus-Experienced CD8+ and CD4+CD8+ T Cells
Source: Front Immunol. 2019 Jan 17;9:3188. doi: 10.3389/fimmu.2018.03188 (PMC6344446; doi:10.3389/fimmu.2018.03188)
Supplement: Supplementary file 1 [file Data_Sheet_1.PDF]

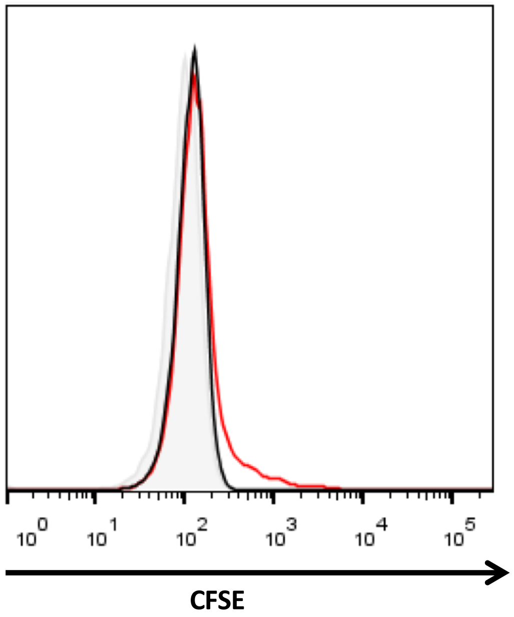

Supplemental Figure 1: NK cells do not take up free CFSE from lysed K562 cells which has not covalently bound to cellular proteins

Histogram shows the CFSE signal of IL-2-primed NK cells that were incubated for 2h with CFSE-labeled K562 cells (red open histogram), for 2 h with supernatant of CFSE-labeled K562 cells that had been incubated before for 2h with NK cells to trigger K562 cell killing (black open histogram) or with no target cells (grey shaded histogram).
