# Supplementary material for: Porcine NK Cells Stimulate Proliferation of Pseudorabies Virus-Experienced CD8+ and CD4+CD8+ T Cells
Source: Front Immunol. 2019 Jan 17;9:3188. doi: 10.3389/fimmu.2018.03188 (PMC6344446; doi:10.3389/fimmu.2018.03188)
Supplement: Supplementary file 2 [file Data_Sheet_2.PDF]

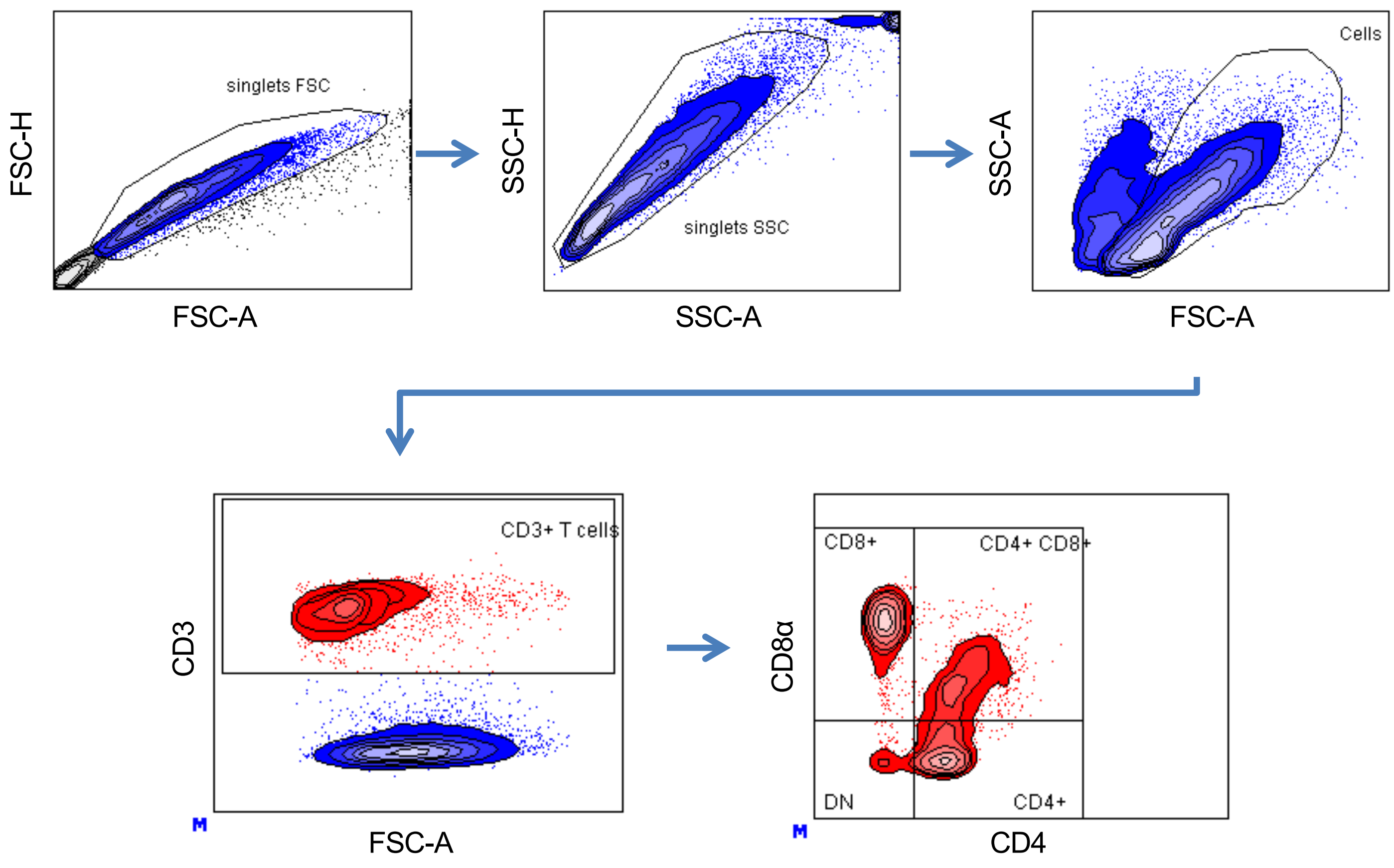

Supplemental Figure 2: Gating strategy of T cell subsets

Doublets were excluded based on their forward scatter and side scatter pattern. NK and T cells were gated according to their light scatter properties. Staining for CD3, CD8α and CD4 was performed and cells were analyzed by flow cytometry. Porcine T cells are characterized by $CD3^{+}$ expression. Different T cell subsets were discriminated based on their CD4 and CD8α expression.
