# Supplementary material for: Porcine NK Cells Stimulate Proliferation of Pseudorabies Virus-Experienced CD8+ and CD4+CD8+ T Cells
Source: Front Immunol. 2019 Jan 17;9:3188. doi: 10.3389/fimmu.2018.03188 (PMC6344446; doi:10.3389/fimmu.2018.03188)
Supplement: Supplementary file 3 [file Data_Sheet_3.PDF]

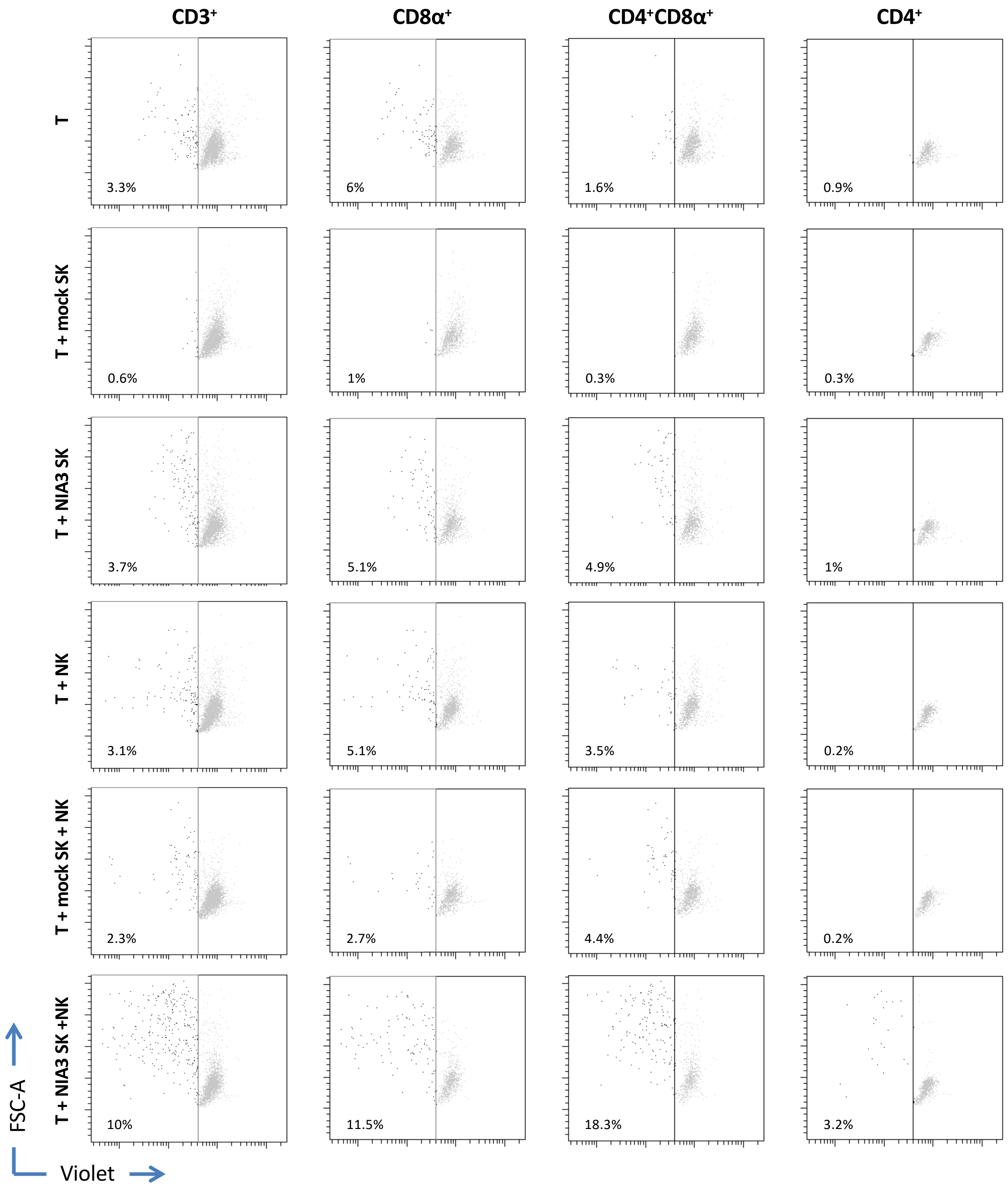


**Supplemental Figure 3: Violet proliferation dot plots of PRV-vaccinated animal**

Dot plots of one PRV-vaccinated animal (out of three) show fluorescence signals of violet dye-labeled T cells/T subsets that were cultured alone or in the presence of NK cells and/or mock- or PRV-infected SK cells (mock SK or NIA3 SK, respectively) for 4 days.
